# Integrated Transcriptomic and Physiological and Biochemical Analysis Revealed Response Mechanism of Rice (*Oryza sativa* L.) to Methylmercury Toxicity Stress

**DOI:** 10.3390/toxics13110989

**Published:** 2025-11-17

**Authors:** Lin Liu, Kang Wang, Shuiting Long, Wentao Li, Adili Batuer, Lei Wang, Tinjia Ma, Xiaohang Xu, Longchao Liang, Md. Habibullah-Al-Mamun, Guangle Qiu

**Affiliations:** 1 College of Civil Engineering and Architecture, Xinjiang University, Urumqi 830047, China; wangkang@stu.xju.edu.cn (K.W.); 107552404605@stu.xju.edu.cn (W.L.); m13162200236_1@163.com (A.B.); 13919203389@163.com (T.M.); 2 Disease Prevention and Control Center of Qiandongnan Miao and Dong Autonomous Prefecture, Kaili 556099, China; 13385153995@163.com; 3 College of Environmental Science and Engineering, Institute of Pollution Control and Ecological Safety, Tongji University, Shanghai 200092, China; celwang@tongji.edu.cn; 4 Key Laboratory of Karst Georesources and Environment, Ministry of Education, College of Resources and Environmental Engineering, Guizhou University, Guiyang 550025, China; xuxh@gzu.edu.cn; 5 School of Chemistry and Materials Science, Guizhou Normal University, Guiyang 550025, China; lianglc139@gznu.edu.cn; 6 Department of Fisheries, Faculty of Biological Sciences, University of Dhaka, Dhaka 1000, Bangladesh; almamunhabib@du.ac.bd; 7 State Key Laboratory of Environmental Geochemistry, Institute of Geochemistry, Chinese Academy of Sciences, Guiyang 550081, China

**Keywords:** methylmercury (MeHg), rice, miRNA, physiological and biochemical responses, high-throughput sequencing

## Abstract

Methylmercury (MeHg), a pervasive environmental contaminant, poses significant human health risks due to its bioaccumulation in food chain, particularly through rice, a dietary staple for billions of people. Although extensive research has been conducted on the environmental cycling and health impacts of MeHg on rice, limited attention has been given to understanding the molecular and physiological responses to MeHg stress, which is crucial for elucidating the mechanisms of detoxification and adaptation. Herein, we conducted pot experiments of rice with varying MeHg concentrations of soil, followed by high-throughput sequencing and assessment of physiological and biochemical responses in order to evaluate the impacts of MeHg exposure on rice growth, stress tolerance, and underlying molecular mechanisms. The results showed that significant increases in root-to-stem translocation of MeHg occurred, further inducing oxidative stress, as evidenced by alterations in antioxidant enzyme activities (CAT, POD, and SOD), proline (PRO) content, and chlorophyll levels, resulting in cellular damage and stunted plant growth. Transcriptome analysis identified differentially expressed miRNAs (DE-miRNAs) in rice roots associated with metabolic regulation, signal transduction, biosynthesis, and plant–pathogen interactions. Notably, genes involved in starch and sucrose metabolism, the Target of Rapamycin (TOR) signaling pathways, and phenylpropanoid biosynthesis were found to be key in rice’s response to MeHg toxicity. Protein–protein interaction (PPI) and miRNA–target gene analyses further highlighted genes encoding jasmonic acid-amido synthetase and FERONIA-like receptors as potential candidates for detoxification mechanisms. This study contributes to building the molecular regulation network and physiological underpinnings of rice’s response to MeHg stress, providing insights into potential targets for genetic improvement to enhance rice’s resilience to MeHg toxicity.

## 1. Introduction

Mercury (Hg), a toxic trace element notorious for its devastating effects on various life forms, presents a substantial threat to human health when excessively accumulated in plants; this concern is heightened by its biomagnification in the food chain [1]. The toxicity of Hg is predominantly influenced by its chemical form, with methylmercury (MeHg, CH_3_HgCl) emerging as the primary concern due to its toxicity to humans and its exceptional capacity for bioaccumulation as it can cause neurological damage, especially in fetuses and young children, impairing cognitive and motor functions [2]. Rice (*Oryza sativa* L.), as a significant accumulator of MeHg in its grains from soil, serves as a staple food for over half of the global population. In Europe, MeHg concentrations in agricultural soils vary between 3 and 1600 μg kg^−1^, while in India and Indonesia, the concentrations can reach up to 580 and 8364 μg kg^−1^, respectively [3,4,5]. Notably, in the Wanshan region of China, MeHg concentrations can reach 44 μg kg^−1^ in rice cultivated on MeHg-contaminated soils [6]. Therefore, to mitigate MeHg accumulation in rice, it is crucial to investigate the responses and underlying mechanisms of MeHg transport in rice.

Once under MeHg stress, rice undergo a range of physiological and biochemical variation, including increased production of reactive oxygen species (ROS) and enhanced antioxidant enzyme activity which serve as indicators of oxidative stress and cell membrane damage [7], and the excessive accumulation of ROS can cause cellular damage and indirectly stunt plant growth [8,9]. Correspondingly, plants have developed various antioxidant defense strategies to counteract the harmful effects of MeHg, including non-enzymatic antioxidants such as glutathione (GSH) and ascorbic acid which chelate MeHg ions, along with antioxidant enzymes like superoxide dismutase (SOD), peroxidase (POD), and catalase (CAT) which are responsible for scavenging excess ROS [10,11]. For instance, CAT expression and activity are useful markers for assessing aluminum (Al) resistance in soybeans [12], while SOD, CAT, and POD activity measurement can reflect antioxidant capacity in raspberry fruits [13]. However, relying solely on limited detection methods may lead to significant errors, and focusing on a single plant organ fails to provide a comprehensive understanding of the complex interactions and mutual influences among different plant parts under MeHg stress.

In addition, the accumulation of malondialdehyde (MDA) can serve as a reliable indicator of MeHg stress, as it results from the generation of superoxide radicals which further trigger lipid peroxidation in cellular membranes [14,15]. MeHg stress also induces increased proline (PRO) synthesis, which enhances stress tolerance by preserving cell turgor, maintaining osmotic balance, and regulating ROS levels to prevent oxidative damage [16]. The photosynthetic efficiency of above-ground leaves is also significantly impacted by MeHg stress, as rice roots tend to accumulate the majority of the MeHg, preferentially sequester dry matter accumulation and ultimately reducing crop yield [17]. MDA, PRO, and chlorophyll (CHL) levels can be employed to validate changes in the activity of key antioxidant enzymes such as SOD, CAT, and POD, further confirming the physiological and biochemical changes occurring in various rice organs under MeHg stress. Nevertheless, physiological and biochemical indicators have certain limitations in reflecting the sensitivity, subtle changes, and early damage caused by MeHg stress, lacking in-depth analysis of the gene regulation, signal transduction, and molecular mechanisms triggered by MeHg stress.

High-throughput RNA transcriptome sequencing (RNA-Seq), with its advantages of high sensitivity, specificity, and throughput, can precisely analyze miRNA regulatory networks, providing more comprehensive data support for studying molecular response mechanisms under MeHg stress [18,19]. Transcriptome profiling has identified MeHg-responsive genes in rice roots involved in glutathione metabolism, amino acid biosynthesis, secondary metabolite production, water transport, and detoxification under MeHg stress [11,20,21]. Notably, genes involved in the synthesis of phenylalanine, tyrosine, and tryptophan are upregulated in response to MeHg stress [11]. This upregulation promotes the production of phenylpropanoids in rice roots, which enhances cellular structure, inhibits pathogen invasion, and significantly reduces MeHg-induced ROS generation, thereby improving rice tolerance to MeHg [20,22]. Moreover, transcriptomic analyses have shown that genes commonly upregulated under MeHg stress are associated with chelation and hormonal regulation, cellular structure, lipid secondary metabolism, and carbohydrate metabolism [23,24]. Although plenty of physiological evidence has revealed the importance of antioxidative systems in MeHg tolerance for rice, investigations are still limited in term of the molecular mechanisms and transcriptome response—especially regarding the key genes and pathways of the detoxification response to MeHg stress in rice. Furthermore, the studies above primarily concentrated on transcriptomic analyses of rice seedling roots exposed to low-MeHg concentrations under short-term hydroponic conditions. There remains a significant gap in understanding how rice responds to prolonged exposure to varying levels of MeHg-contaminated soils, particularly regarding the underlying mechanisms of response to MeHg stress.

Herein, rice plants were exposed to MeHg stress in soil for 60 days, spanning critical growth phases from the seedling stage to the booting and jointing stages which are essential for root maturation and the transition from vegetative growth to reproductive development. This period was selected as it is particularly significant for MeHg uptake, making it an ideal timeframe to investigate root responses to MeHg stress. Firstly, we assessed the impact of varying levels of MeHg stress on rice growth and measured MeHg concentrations in the roots, stems, and leaves at the post-exposure stage. Subsequently, transcriptomic analyses were performed on the roots to identify and compare genetic responses across different MeHg stress levels, aiming to pinpoint critical genes and pathways involved in rice’s adaptive mechanisms to MeHg. Finally, physiological and biochemical markers in the stems and leaves were analyzed to assess their responses to MeHg exposure. The results are anticipated to provide a comprehensive understanding of rice’s responses to MeHg stress, integrating physiological and biochemical alterations with the underlying molecular mechanisms across varying stress intensities.

## 2. Materials and Methods

### 2.1. Plant Culture and Pot Experiment

The soil used in the pot experiment was collected from the suburb of Huaxi, Guiyang, Guizhou, China, with average MeHg and Hg concentrations of 0.229 and 164 μg kg^−1^, air-dried, and sieved with a 10 (2 mm) mesh for rice culture. A total of 10 kg sieved soil was packed into each plastic pot (36 cm (diameter) × 45 cm (height)). According to the literature on the enrichment of MeHg and pot experiments of MeHg stress in rice [25,26,27,28], the concentration gradients of MeHg were set as 0, 50, 150, and 300 μg kg^−1^ marked as the control (con), low (low), middle (middle) and high (high) dose groups, respectively. Reagent equilibrium was established for 30 days, and there were three pots in each group and three rice plants in each pot. Taking three-line indica hybrid rice Yiyou 1988 (*Oryza sativa* L.), a dominant rice in Guizhou Province, as the test material, seedlings with the same growth were selected and transplanted into pots after the seedlings grew to three leaves. Urea and compound fertilizer were applied to the pot as the base fertilizer. All measurements used the pot as the experimental unit. A single biological replicate was constituted by pooling the three plants from one pot, with three replicates (n = 3) per treatment. Data are presented as the mean ± SD (n = 3 biological replicates).

Rice was stressed with methylmercury chloride (CH_3_HgCl, MeHgCl) for 60 days, and samples of each stress group were collected at the booting and jointing stage, rinsed three times with deionized water, and then 1 × PBS prepared with RNase-free was used to quickly clean the tissue surface and blot the surface liquid. The rice root apical part (3–5 cm) was collected, put into the frozen tube for liquid nitrogen freezing, and stored in a −80 °C freezer. The remaining roots, stems, and leaves were separated and packaged and stored in a −80 °C freezer.

### 2.2. MeHg Analysis

Fresh rice tissue (root, stem, and leaf) was freeze-dried, and rice samples were ground and sieved (200-mesh). In total, 0.1 g rice tissue was accurately weighed and placed in a 50 mL centrifuge tube, and 5 mL of KOH solution (25%) was added to the centrifuge tube which was placed in a water bath at 75 °C for 3 h to fully digest the sample. The tube was shaken every 30 min during the digestion to fully mix the digestion solution and the sample. Concentrated HCl was used to adjust the pH of the digestion solution to acidic. A total of 10 mL of extractant dichloromethane (CH_2_Cl_2_) was added, shaken for 30 min, and centrifuged. The extract was transferred and quantified accurately. The MeHg in the above extract was reverse-extracted to the aqueous phase. The appropriate amount of the reverse extract was determined by gas chromatographic cold vapor atomic fluorescence spectrometry (GC-CVAFS) [29].

### 2.3. Transcriptome Analysis by RNA Sequencing and Quantitative Real-Time PCR (qRT–PCR)

The rice roots were collected from each bioreplicate of various treatment, washed with deionized water, and then crushed with liquid nitrogen. The total RNA of the rice root stressed by MeHg was isolated using TRIzol reagent (Invitrogen, Carlsbad, CA, USA). A sample of 3 μg of total RNA was ligated to sequencing junctions using the TruseqTM Small RNA Sample Preparation Reagent Kit (Illumina, San Diego, CA, USA). Subsequently, cDNA was synthesized by reverse transcription and amplified by PCR cycles to generate libraries for sequencing using the Illumina Hiseq X Ten. The integrity and high quality of RNA were checked using an Agilent 2100 bioanalyzer (Agilent Technologies, Santa Clara, CA, USA), and the quality and concentration of the RNA samples are shown in Table S1. The miRNA expression levels were quantified using TPM (transcripts per kilobase of exon model per million mapped reads) values, and the read counts were subjected to statistical analysis using DESeq2 software (version 1.38.3) based on a negative binomial distribution in order to identify miRNAs with significant expression differences between groups. The fold change (FC) < 0.5 (*p* < 0.05) and the FC values ≥ 2.0 (*p* < 0.05) were set as the threshold for significantly differential expression of genes. We created three differentially expressed miRNA (DE-miRNA) datasets: con vs. low (50 μg kg^−1^ MeHgCl compared to control), con vs. middle (150 μg kg^−1^ MeHgCl compared to control), and con vs. high (300 μg kg^−1^ MeHgCl compared to control).

Target genes of differentially expressed miRNAs were predicted using psRobot_v1.2. A stringent penalty score cutoff of ≤2.5 was employed. All other parameters were set to their software defaults.

RNA was reverse-transcribed and the obtained cDNA was used for qRT-PCR amplification using gene-specific primers (Table S2) and SYBR Green Master Mix (GenStar Biosolutions Co., Ltd., Beijing, China). The 2^−ΔΔCt^ method was applied to calculate the relative expression of specific genes [30]. Detailed information of the total RNA extraction, bioinformatics analysis, and qRT–PCR mentioned above is described in the Supplementary Materials.

### 2.4. Determination of Growth Parameters and Physiological and Biochemical Indices

The physiological and biochemical indices in the roots, stems, and leaves, including the concentrations of total protein (TP), SOD, CHL, MDA, CAT, POD, and PRO, were also determined using reagent kits according to the instructions. Detailed information of the reagent kits for the physiological and biochemical indices mentioned above is described in the Supplementary Materials.

### 2.5. Quality Control and Data Analysis

The quality control of experimental data was carried out by a blank experiment, determination of reference material, and parallel samples. In the determination of MeHg, TORT-3 (Lobster Hepatopancreas Reference Material for Trace Metals) was used as the standard substance, with a calibration value of 137 ± 12 μg kg^−1^ and a recovery rate of 90–110%. The statistical significance of each parameter was determined by one-way analysis of variance (one-way ANOVA). The assumptions of normality (assessed using Shapiro–Wilk test) and homogeneity of variances (assessed using Levene’s test) were evaluated for all datasets subjected to ANOVA. For one-way ANOVA followed by post hoc multiple comparisons, we used the Tukey’s Honest Significant Difference (HSD) test. The data are presented as the mean ± standard deviation (SD) (n = 3 biological replicates).

## 3. Results and Discussion

### 3.1. MeHg Distribution in Rice Under Different Levels of MeHg Stress

**Figure 1:**
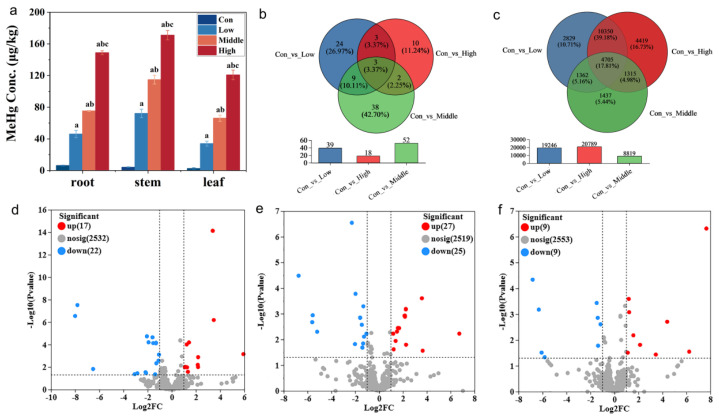
MeHg accumulation of six-week-old rice seedlings after exposure to 50 (low), 150 (middle), and 300 (high) μg kg^−1^ CH_3_HgCl (MeHg) for 60 days and in the control without MeHg addition (CK). (**a**) MeHg concentrations in leaves, stems, and roots (different letters indicate significant differences among the four treatments (*p* < 0.05, one-way ANOVA); mean ± SD). (**b**) Venn diagram of the DE-miRNAs in roots of rice after exposure to MeHg and in the control. (**c**) Venn diagram of the target gene in roots of rice after exposure to MeHg and in the control. (**d**–**f**) Volcano plot of the amount of DE-miRNAs upregulated and downregulated in each treatment (con vs. low, con vs. middle, and con vs. high).

The MeHg concentration in the stems of rice was 72.2 μg kg^−1^ under low MeHg stress, increasing to 115 μg kg^−1^ and 171 μg kg^−1^ under middle and high MeHg stress, respectively (Figure 1a). Similarly, the MeHg content in rice roots and leaves ranged from 46.2 to 148.9 and 34.0 to 120.8 μg kg^−1^, both of which were significantly higher than the control group (6.31 μg kg^−1^ in roots and 2.60 μg kg^−1^ in leaves). Notably, MeHg content across all tissues increased with rising stress levels. These results indicate a shift in MeHg distribution patterns, while the control group followed the sequence root > stem > leaf; under MeHg stress, the distribution shifted to stem > root > leaf. Compared with roots, the higher MeHg concentrations in the stems under MeHg stress should be ascribed to a demethylation phenomenon in roots which is the main occurrence site exposed to MeHg, but this phenomenon was not observed in the control (no MeHg addition) [31]. However, the potential contribution of in planta demethylation to this distribution pattern requires further validation through direct measurement of inorganic Hg in future studies.

To further investigate MeHg migration within rice, the translocation factor (TF) between different plant parts was calculated. The root-to-stem TF of MeHg was 0.672 in the control group but increased significantly under MeHg stress, reaching 1.56, 1.52, and 1.15 for low, middle, and high stress levels, respectively. This suggests enhanced translocation of MeHg from roots to stems under stress conditions [32]. In contrast, no significant differences were observed in the stem-to-leaf TF between the control and stress groups.

To elucidate the molecular basis of MeHg distribution and translocation mechanisms, transcriptomic responses were subsequently investigated.

### 3.2. Transcriptome Response in Roots to MeHg Stress

#### 3.2.1. RNA-Seq Data Quality and DE-miRNAs

Transcriptome sequencing of rice roots generated a total of 226.7 million clean data, with an average of ≥16.09 million reads per sample. The clean datasets exhibited high quality, with an average Q30 base percentage of ≥97.25%, and 25.3–49.3% of clean reads in each sample successfully mapped to the reference genome (Table S3). To evaluate data reliability, inter-sample Venn analysis, correlation analysis, and principal component analysis (PCA) were performed. The Venn analysis revealed 423, 859, 264, and 360 unique miRNAs in the con and low-, middle-, and high-stress groups, respectively, with 445 miRNAs shared across all groups (Figure S1a). Correlation analysis among biological replicates yielded values ranging from 0.775 to 0.903, confirming the consistency of the experimental design and inter-sample relationships (Figure S1b). PCA, based on miRNA expression levels, grouped the samples into distinct clusters, with high similarity observed between the middle- and high-stress groups after dimensionality reduction (Figure S1c,d). These results indicate that the RNA-Seq data were of high quality and suitable for downstream bioinformatics analysis.

Venn analysis of DE-miRNAs and their target genes identified common and unique components across the datasets. In the comparisons between the con vs. low, con vs. middle, and con vs. high datasets, 19,246, 8819, and 20,789 target genes exhibited significantly differential expression; among these, 2829, 1437, and 4419 target genes were specifically expressed in the respective datasets, and a total of 4705 target genes were shared across all comparisons (Figure 1c). In terms of DE-miRNAs, 39, 52, and 18 DE-miRNAs were identified in the con vs. low, con vs. middle, and con vs. high datasets, respectively (Figure 1b). Among these, 24, 38, and 10 DE-miRNAs were unique to each comparison, while 3 DE-miRNAs were shared across all datasets, including 2 upregulated DE-miRNAs (osa-miR319a-3p and osa-miR390-5p) and 1 downregulated miRNA (osa-miR156b-3p) (Figure 1b). Volcano plots further illustrated that most DE-miRNAs were downregulated in each dataset. Specifically, in the con vs. low datasets, 17 DE-miRNAs were upregulated and 22 DE-miRNAs were downregulated; in the con vs. middle datasets, 27 DE-miRNAs were upregulated and 25 DE-miRNAs were downregulated; and in the con vs. high datasets, 9 DE-miRNAs were upregulated and 9 DE-miRNAs were downregulated (Figure 1d–f).

**Figure 2:**
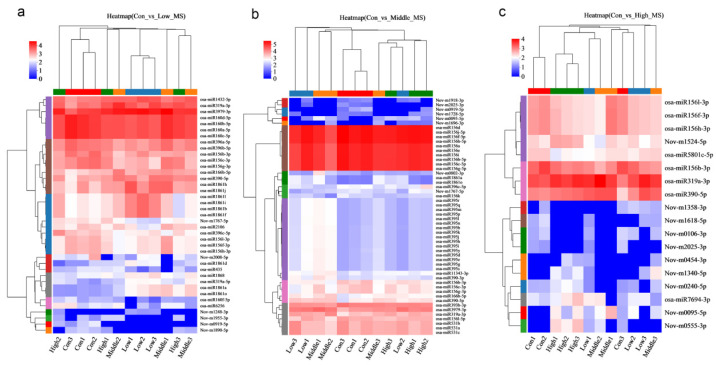
Hierarchical cluster heatmaps showing that 39, 52, and 18 genes were upregulated and downregulated expression under (**a**) low, (**b**) middle, and (**c**) high MeHg stress compared with the control treatment.

To further investigate differences in MeHg tolerance, gene expression patterns in the three stress groups were analyzed. The hierarchical cluster heatmap revealed that 39, 52, and 18 DE-miRNAs were upregulated or downregulated under low, middle, and high MeHg stress, compared to the control group, respectively (Figure 2). In the con vs. low datasets, the miRNA1861 family (9 members) and the miRNA319 family (2 members) were upregulated, which contrasts with the downregulation of osa-miR1861a in rice under arsenic stress [33]. This difference in expression patterns may indicate the distinct roles played by miRNA family members and emphasize the complex regulatory mechanisms in rice responding to various heavy metal stressors. The miRNA156 family (6 members) and the miRNA160 family (5 members) are downregulated. In the con vs. middle datasets, several miRNA families were significantly upregulated, including the miRNA395 family (16 members), miRNA1861 family (2 members), miRNA390 family (2 members), and miRNA393b-3p (1 member). This is consistent with the upregulation of miR393 in Medicago truncatula under Hg stress, suggesting that these miRNAs may alleviate Hg toxicity by regulating target genes expression [34]. Conversely, the miRNA156 family (15 members) and miRNA531 (3 members) were downregulated. In the con vs. high datasets, osa-miR319a-3p and osa-miR390-5p were upregulated, while the miRNA156 family (4 members) was downregulated under MeHg stress (Figure 2).

These results highlight the dynamic regulation of miRNAs under varying levels of MeHg stress and suggest that specific miRNA families play critical roles in rice’s adaptive response to MeHg toxicity. To further elucidate the underlying molecular mechanisms of MeHg stress tolerance, functional annotation and enrichment analysis of the DE-miRNAs were subsequently performed.

#### 3.2.2. Gene Ontology (GO) and Kyoto Encyclopedia of Genes and Genomes (KEGG) Enrichment of DE-miRNAs

Functional annotation of target genes was performed using six databases (NR, Swiss-Prot, EggNOG, GO, KEGG, and Pfam) through the plant prediction software psRobot_v1.2 (Figure S2a). The upregulated miRNA predicted 464 target genes, while downregulated miRNA predicted 357 target genes. In the con vs. low, con vs. middle, and con vs. high groups, different miRNAs predicted a total of 285, 537, and 426 annotated target genes, respectively (Table S4). These results indicate that a significant number of target genes, which are regulated by the DE-miRNAs, are crucial in mediating the rice stress response.

**Figure 3:**
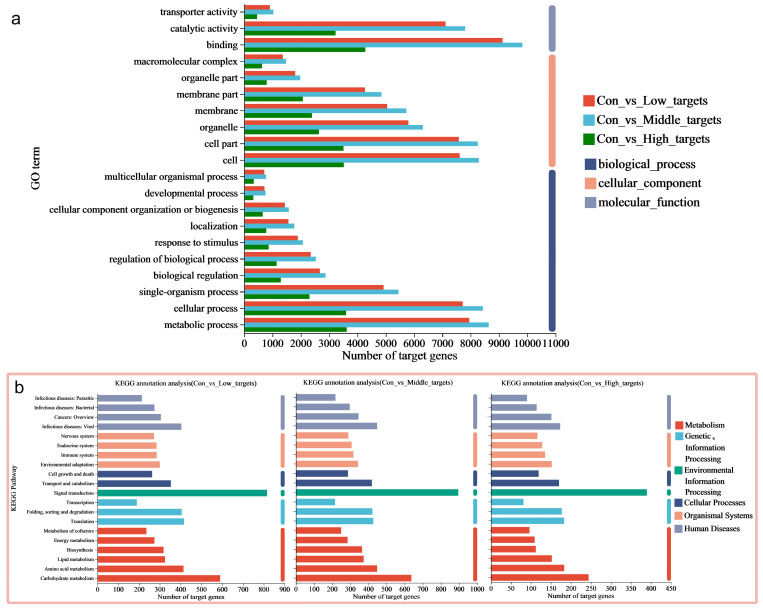
(**a**) Gene Ontology (GO) and (**b**) Kyoto Encyclopedia of Genes and Genomes (KEGG) annotation analysis showing that multiple terms/pathways were enriched in the 19,246, 8819, and 20,789 miRNAs shared by the three datasets, i.e., con vs. low, con vs. middle, and con vs. high.

**Figure 4:**
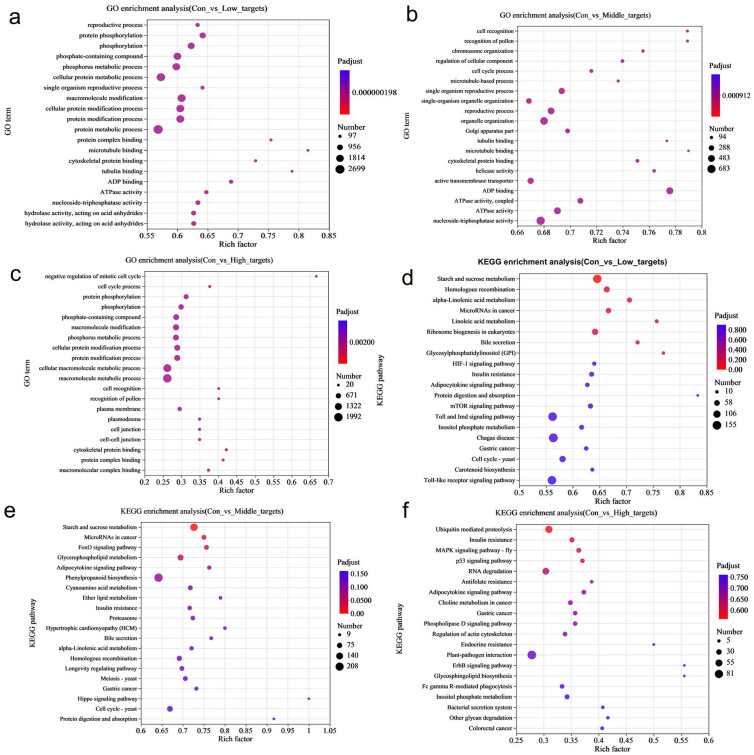
(**a**–**c**) GO and (**d**–**f**) KEGG enrichment analysis indicated that the terms/pathways of rice roots under MeHg stress. The top 20 most enriched GO terms/KEGG pathways are displayed, with the size of each circle representing the miRNA number enriched in individual GO terms/KEGG pathway and the rich factor in the x-axis representing how significant the degree of enrichment is in the pathway.

In order to further investigate the biological functions of DE-miRNAs, potential target genes were classified using GO terms. Fisher’s exact test revealed significant enrichment of the GO terms (adjusted *p*-value (Padjust) < 0.05), highlighting the regulatory role of DE-miRNAs in rice under MeHg stress (Table S5). The annotation analysis identified multiple GO terms enriched in 39, 52, and 18 DE-miRNAs in the three datasets (con vs. low, con vs. middle, and con vs. high). These enriched terms include biological processes (particularly metabolism and cellular processes), cellular components (especially cell and cell part), and molecular functions (primarily binding and catalytic activity) (Figure 3a and Table S5). Further enrichment analysis indicated that the key terms for rice roots under MeHg stress include metabolic processes (especially protein and cellular protein metabolism), modifications (particularly protein, macromolecule, and cellular protein modifications), and phosphorylation (including phosphorus and phosphate-containing compound metabolism, as well as protein phosphorylation) (Figure 4a–c and Table S5). Therefore, the potential target genes of the DE-miRNAs in rice may influence the components of the plasma membrane, participate in cellular protein metabolism, protein phosphorylation, and protein modification, and be associated with phosphorus metabolism, ADP binding, and nucleoside triphosphatase activity.

Furthermore, target genes were classified based on their involvement in specific pathways or functions using the KEGG database. The annotation analysis revealed that multiple pathways were enriched in the target genes shared partly across the three datasets, including metabolic pathways (carbohydrate and amino acid metabolism), genetic information processing (translation and folding; sorting and degradation), environmental information processing (signal transduction), cellular processes (transport, catabolism, and cell growth and death), and organismal systems (environmental adaptation and immune system) (Figure 3b and Table S6). The enrichment analysis further showed that the key pathways for rice roots under MeHg stress include metabolic pathways (starch and sucrose metabolism, inositol phosphate metabolism, and glycerophospholipid metabolism), signaling pathways (Target of Rapamycin (TOR) signaling pathways), biosynthesis, and plant–pathogen interactions (phenylpropanoid biosynthesis and plant–pathogen interaction) (Figure 4d–f and Table S6).

The target genes are primarily enriched in biosynthesis, metabolic processes, and signal transduction pathways, indicating that these biological processes play a crucial role in rice’s resistance to MeHg stress. KEGG pathway analysis further indicates that rice may mitigate MeHg toxicity through the interaction of multiple target genes and pathways.

### 3.3. Specific Responsive Genes Under MeHg Stress

#### 3.3.1. Metabolism-Related Genes

Numerous genes involved in metabolism-related processes were identified, encoding starch and sucrose metabolism, inositol phosphate metabolism, cyanoamino acid metabolism, glycerophospholipid metabolism, and others (Table S6).

In the con vs. low and con vs. middle datasets, the starch and sucrose metabolism pathway (map00500) was associated with 144 and 162 genes, respectively (Table S6), indicating that MeHg stress perhaps disrupts rice photosynthesis and carbohydrate metabolism, specifically the synthesis and degradation of starch and sucrose. MiRNAs are thought to regulate the accumulation and distribution of these carbohydrates by modulating the expression of genes involved in starch and sucrose synthesis and related transporters [35]. This regulation is essential for maintaining energy homeostasis in rice under MeHg stress. Inositol phosphate metabolism (pathway map00562) showed a gene enrichment of 45 and 25 in the con vs. low and con vs. high datasets, respectively (Table S6). Phosphoinositides, key components of cell membranes, play critical roles in signal transduction and cellular response mechanisms, which indicates that rice may regulate phosphoinositide metabolism to maintain membrane stability and function under MeHg stress. MiRNA could influence the synthesis and degradation of phosphoinositides by modulating the expression of key enzymes (phosphoinositide synthase and phosphatase), thereby regulating rice growth and development in response to MeHg stress [36]. The gene enrichment for glycerophospholipid metabolism (pathway map00564) in the con vs. middle dataset was 100 (Table S6). Glycerophospholipids are also essential components of cell membranes, crucial for maintaining membrane integrity and fluidity. MiRNAs may regulate key enzymes involved in the synthesis and degradation of glycerophospholipids, thereby aiding in the repair and protection of cell membranes under MeHg stress [37]. Cyanoamino acid metabolism (pathway map00460) exhibited a gene enrichment of 61 in the con vs. middle dataset (Table S6). This pathway is closely linked to nitrogen metabolism and detoxification mechanisms in rice [38], indicated that miRNAs may influence cyanoamino acid accumulation and detoxification by regulating the expression of key enzymes involved in cyanoamino acid synthesis and degradation [39].

#### 3.3.2. Signal Transduction-Related Genes

Numerous miRNAs enriched in signal transduction pathways were identified as regulating TOR, among others (Table S6).

The TOR signaling pathway (map04150) was associated with the enrichment of 50 genes in the con vs. low dataset (Table S6). The processes of rice growth and development, metabolism, and stress resistance are largely mediated by the regulation of TOR expression [40]. TOR may facilitate cellular adaptation by regulating autophagy, protein synthesis, and energy metabolism, thereby mitigating MeHg’s toxic effects [41]. MeHg-induced cytotoxicity likely inhibits TOR kinase activity. This inhibition would relieve the brake on the autophagy initiation complex, thereby inducing autophagosome formation; the activation of autophagy plays a dual role, acting as a pro-survival mechanism by clearing damaged components, or triggering cell death if excessively sustained. Therefore, the functional outcome of the TOR–autophagy axis is critical for determining a plant’s tolerance to MeHg [42]. Consequently, specific receptors can detect stress resistance molecular patterns, thereby activating the signaling pathway and triggering the rice defense response under MeHg stress in rice.

#### 3.3.3. Biosynthesis and Plant–Pathogen Interaction Related Genes

Multiple enriched genes in rice roots were identified as being associated with biosynthesis (Table S6). Notably, a significant portion of these genes are involved in phenylpropanoid biosynthesis, plant–pathogen interactions, and related processes. A total of 208 genes associated with phenylpropanoid biosynthesis (pathway map00940) were enriched in the con vs. middle dataset (Table S6). This pathway, a cornerstone of secondary metabolism, produces a variety of secondary metabolites of phenylpropanoids, lignin, and flavonoids by enzymatic reactions. These metabolites may play pivotal roles in detoxification and antioxidant defense under MeHg stress [20]. The phenylpropanoid pathway in rice roots may be activated by MeHg stress, reinforcing plant cell walls to prevent pathogen colonization and mitigating the toxic effects of MeHg [22]. In addition, 81 genes related to plant–pathogen interaction (pathway map04626) were enriched in the con vs. high dataset (Table S6). The plant–pathogen interaction is inherently complex, involving the activation of the plant immune system and the deployment of defense to resist pathogen infection, could profoundly influence plant growth and development under MeHg stress.

We observed that metabolism, biosynthesis, and signal transduction pathways exhibited heightened activity in response to MeHg stress in rice. These pathways are likely integral to MeHg tolerance and detoxification in rice. Notably, the regulatory role of miRNAs is typically not standalone but functions in conjunction with other regulatory mechanisms, including transcription factors and protein interactions. Thus, metabolic regulation in cells under MeHg stress represents a multifaceted and highly coordinated process.

### 3.4. Protein–Protein Interaction (PPI) Analysis of the Commonly Responsive DE-miRNAs

PPI network analysis of the commonly responsive DE-miRNAs identified key hub genes (Figure S2b) which are likely associated with the response to MeHg stress. These genes include BGIOSGA034955, BGIOSGA012829, BGIOSGA017715, BGIOSGA013411, BGIOSGA000306, BGIOSGA018821, BGIOSGA007312, BGIOSGA003240, BGIOSGA012197, BGIOSGA006922, and BGIOSGA017550 (Table S7). Among them, BGIOSGA012829 encodes a transcription-associated DNA-dependent RNA polymerase which uses ribonucleoside triphosphates as substrates to catalyze the transcription of DNA into RNA, thus contributing to resistance to stress [43]. Specifically, the expression of the BGIOSGA034955 gene was significantly upregulated under stress. This gene encodes jasmonic acid-amido synthetase (JAR1), which plays a critical role in enhancing rice’s resistance [44]. Additionally, BGIOSGA017715, BGIOSGA013411, and BGIOSGA000306 encode FERONIA-like receptor (FLR) genes which facilitate ROS production and enhance defense-related gene expression in response to Pyricularia oryzae in Arabidopsis thaliana [45].

### 3.5. miRNA–Target Gene Correspondence Analysis of the Commonly Responsive DE-miRNAs

Table S8 presents the expression levels [log_2_(fold change)] of the commonly responsive DE-miRNAs for several miRNAs, including osa-miR156b-3p, osa-miR319a-3p, and osa-miR390-5p. Furtherly, a total of six crucial hub genes were identified through miRNA–target gene correspondence analysis of the commonly responsive DE-miRNAs (Figure S2c). These genes are likely associated with the response to MeHg stress, including BGIOSGA000900, BGIOSGA030834, BGIOSGA003398, BGIOSGA008799, BGIOSGA018043, and BGIOSGA025466 (Table S9).

Among these, BGIOSGA013525 and BGIOSGA003398 encode lipoxygenase and peroxidase, enzymes critical for mitigating oxidative damage by preventing lipid peroxidation and mitigating stress. Furthermore, BGIOSGA000900, BGIOSGA030834, and BGIOSGA025466 encode starch and cellulose synthases, which are involved in host defense response and susceptibility to Rhizoctonia solani in rice. Lastly, the expression of BGIOSGA018043, which encodes an MLO-like protein, is associated with rice blast fungus infection under MeHg stress [46].

### 3.6. qRT-PCR Validation

**Figure 5:**
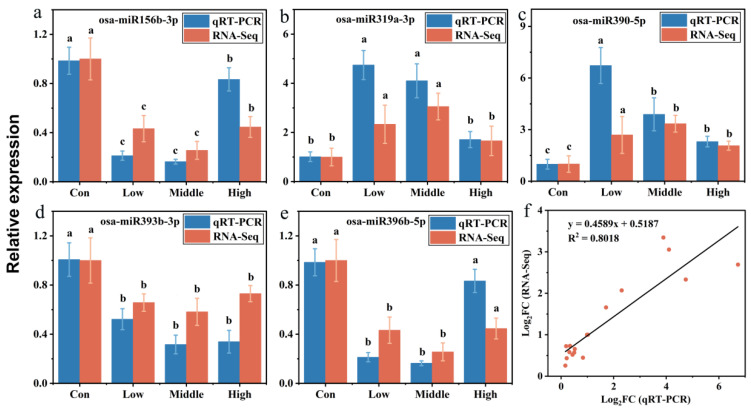
qRT-PCR validation of miRNA. The relative expression of specific miRNAs was calculated using the 2^−ΔΔCt^ method. Five miRNAs found to be differentially expressed were analyzed by qRT-PCR. (**a**) Metabolism processes-related miRNA. (**b**) Signal transduction-related miRNA. (**c**) Biosynthesis and plant–pathogen interaction-related miRNA. (**d**,**e**) Two miRNAs related to heavy metals stress were screened. (**f**) Correlation analysis of gene expression between RNA-Seq data and qRT-PCR results. RNA-Seq data were plotted against data from qRT-PCR. Data are presented as a [log_2_(fold change)] value (log_2_FC). Different letters (a, b, c) above the bars indicate statistically significant differences (*p* < 0.05, one-way ANOVA followed by Tukey’s HSD test).

qRT–PCR was employed to verify the expression levels of upregulated (osa-miR319a-3p, and osa-miR390-5p) and downregulated miRNA (osa-miR156b-3p, osa-miR393-3p, and osa-miR396b-5p) [47]. The results demonstrated significant upregulation of osa-miR319a-3p, which regulates the mRNA transcripts of genes encoding chlorophyll a-b binding protein, chloroplastic, MLO-like protein, and peroxidase under MeHg stress (Figure 2a). In the biosynthesis and plant–pathogen interaction group, the expression of osa-miR390-5p was significantly upregulated under MeHg stress (Figure 2b). The expression of osa-miR156b-3p, which regulates the mRNA transcripts of genes encoding lipoxygenase, starch synthase, chloroplastic, and cellulose synthase, was significantly downregulated under MeHg stress (Figure 2c). Comparison of miRNA expression detected by high-throughput sequencing and qRT-PCR revealed consistent expression trends, confirming the reliability of the sequencing results (Figure 5). It is important to note that the regulatory relationships between the identified DE-miRNAs and their putative target genes proposed in this study are primarily based on in silico predictions and cross-referencing with existing databases. While we have employed stringent criteria to increase prediction confidence, direct experimental validation, such as 5′-RLM-RACE or degradome sequencing, will be essential in future work to conclusively confirm these interactions under MeHg stress conditions in rice.

RNA-Seq data were further validated by qRT-PCR (Table S8), with the [log_2_(fold change)] values calculated from both methods exhibiting a strong linear regression (y = 0.4589x + 0.5187, R^2^ = 0.8018, Figure 5f). This linear regression analysis underscores the accuracy of the RNA-Seq transcriptome data and the associated bioinformatics analysis. Additionally, the miRNAs validated by qRT-PCR, such as osa-miR319a-3p and osa-miR390-5p which displayed significant responses to MeHg stress, represent promising candidate target miRNAs for further investigation of the detoxification mechanisms in rice.

### 3.7. Physiological and Biochemical Responses in Rice Under MeHg Stress

The TF of root-to-stem MeHg in the low, middle, and high MeHg stress groups (1.56, 1.52, and 1.15, respectively) was significantly higher than that in the control group (0.672), reflecting the activation of physiological and biochemical responses in rice tissues to counteract MeHg stress. Specifically, rice defense mechanisms are triggered, leading to the upregulation of antioxidant enzymes such as CAT, POD, and SOD, alongside non-enzymatic antioxidants like GSH and ascorbic acid (ASA) which play crucial roles in neutralizing ROS, thereby mitigating oxidative damage and maintaining cellular integrity [10,11]. Additionally, PRO and soluble proteins were increased to enhance osmotic regulation and improve stress resistance [48,49].

**Figure 6:**
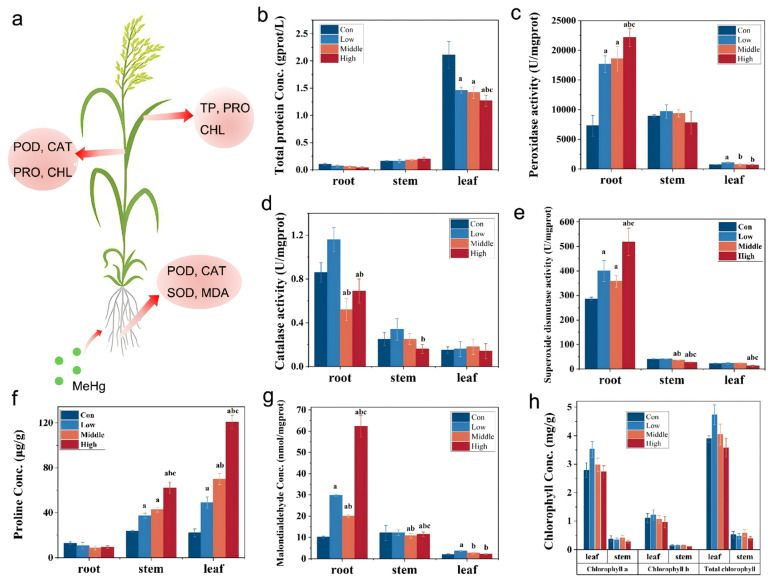
Physiological and biochemical responses of six-week-old rice seedlings after exposure to low, middle, and high MeHg for 60 days and in CK. (**a**) Indices of different parts/organs of rice changed significantly under MeHg stress. (**b**) TP. (**c**) POD. (**d**) CAT, (**e**) SOD, (**f**) PRO, (**g**) MDA, and (**h**) CHL activities. Different letters (a, b, c) above the bars indicate statistically significant differences (*p* < 0.05, one-way ANOVA followed by Tukey’s HSD test).

Although the total protein content in various rice tissues did not exhibit a significant trend across different MeHg dose groups (Figure 6b), leaf tissues displayed the highest protein content (1.27–2.11 gprot/L), significantly exceeding that of the root (0.04–0.10 gprot/L) and stem tissues (0.16–0.20 gprot/L). In the root, the protein content was slightly lower in the MeHg stress groups (0.04–0.07 gprot/L) than in the control group (0.10 gprot/L; *p* < 0.05). In stems, the total protein content was slightly higher in the stress group (0.16–0.20 gprot/L) compared to the control group (0.16 gprot/L; *p* < 0.05). In contrast, the leaf protein content showed a declining trend with increasing MeHg stress, likely influenced by two factors: (i) protein degradation due to denaturation caused by the attachment of MeHg to the sulfhydryl (-SH) groups; (ii) high MeHg doses hindering the synthesis of key plant defense components such as metallothioneins and stress proteins, thereby reducing soluble protein levels [50].

The changes in the protein levels of leaves suggest that MeHg stress may disrupt cellular homeostasis. This disruption is further evident in the activation of antioxidant enzymes such as POD, CAT, and SOD, which participated in scavenging excess H_2_O_2_ and O^2−^ within rice cells and synergistically maintaining membrane stability [13]. A comparison of POD activity across different rice tissues revealed that root activity was significantly higher than that in the stem and leaf (Figure 6c). In the stressed groups, POD activity of root was markedly elevated compared to the control group (*p* < 0.05), showing a positive correlation with increasing MeHg concentration. The highest enzyme activity was observed in the high-stress group, reaching 22.2 ± 1.51 U/gprot, a 204% increase relative to the control group. Interestingly, the POD activity in the stem in the low-stress group exceeded that of the middle- and high-stress groups, as well as the control group (*p* < 0.05). This observation may be attributed to an initial enhancement of antioxidant defense mechanisms at low stress levels, which diminishes under higher stress conditions due to enzyme inhibition caused by oxidative damage. For CAT activity, roots exhibited higher levels compared to stems and leaves. In roots under MeHg stress, CAT activity initially increased and then decreased with rising MeHg concentration (Figure 6d). Notably, the low-stress group displayed elevated CAT activities in both roots (1.16 ± 0.11 U/mgprot) and stems (0.34 ± 0.10 U/mgprot). In contrast, leaves displayed peak CAT activity in the middle-stress group, with a value of 0.18 ± 0.07 U/mgprot. No significant differences in CAT activity were observed among the stress groups in the stem and leaf tissues. Similarly, SOD activity under MeHg stress varied significantly across different rice tissues. Roots exhibited significantly higher SOD activity than stems and leaves (Figure 6e). Roots’ SOD activity was substantially greater than that in the control group (*p* < 0.05) under stress, increasing by 39.87%, 24.97%, and 81.15% in the low-, middle-, and high-stress groups, respectively. No significant differences in SOD activity were found between the stress groups in the stem and leaf tissues. The trends in CAT and POD activity in rice stems and leaves under MeHg stress followed a pattern of low-dose stimulation and high-dose inhibition [51]. This response likely reflects the enhanced activation of defense mechanisms under low MeHg stress, where enzyme activity increases to neutralize ROS and free radicals. However, as MeHg concentration rises, excessive ROS production overwhelms the antioxidant system, leading to enzyme inhibition. In contrast to the responses in the stems and leaves, POD and SOD activities in the roots increase consistently with higher MeHg concentration. This is likely due to the roots being directly exposed to MeHg, triggering robust production of highly active enzymes to mitigate the toxicity. GSH, as a primary chelator, binds to MeHg ions to form less toxic GSH–Hg complexes, which is the first step in cellular detoxification; GSH serves as the substrate for the synthesis of phytochelatins (PCs) which have a higher affinity for heavy metals and are crucial for the sequestration and vacuolar compartmentalization of MeHg/Hg, thereby reducing its toxicity in the cytosol [10,11].

PRO, a crucial osmotic regulator within cells, helps rice adapt to external stress by maintaining osmotic balance, detoxifying ROS, protecting membrane integrity, and stabilizing enzymes and proteins [52]. Compared to the control group (12.8 μg/g), PRO content in roots under MeHg stress was slightly lower (8.69–10.7 μg/g) (Figure 6f). In contrast, PRO content in the stems (37.3–62.1 μg/g) and leaves (48.9–121 μg/g) significantly increased under MeHg stress (23.4 and 21.9 μg/g, in the control group, respectively, *p* < 0.05) (Figure 6f) and exhibited an upward trend with increasing stress doses. These findings suggest that PRO accumulation in the stems and leaves is more sensitive to MeHg stress than in the roots, with lower PRO levels in the roots compared to the leaves under Hg stress [53]. MDA, the end product of lipid peroxidation, is commonly used as an indicator of lipid peroxidation and the tolerance of rice to stress [54]. The MDA content in the roots was significantly higher than in the stems and leaves (Figure 6g), indicating that MeHg stress has a more pronounced effect on lipid peroxidation in the roots. The MDA content in the roots increased significantly with rising MeHg concentrations, ranging from 98.8% to 517% higher than that in the control group (*p* < 0.05), demonstrating a clear dose–response relationship. In the leaves, the MDA content in the middle- and high-stress groups was slightly lower than that in the low-stress group, while no significant differences were observed among the stress groups in the stems (*p* < 0.05). CHL, a crucial component of photosynthesis, plays a key role in determining the photosynthetic rate of rice. In the stems and leaves, the variation in the decrease for CHL b was consistently higher than that for CHL a, indicating that CHL b is more susceptible to MeHg stress (Figure 6h). In the leaves, the levels of CHL a, CHL b, and total CHL increased under low MeHg stress but subsequently decreased as MeHg concentrations rose. This pattern indicates that low MeHg stress may stimulate CHL synthesis, whereas higher concentrations inhibit it. The changes in MDA and CHL content may be attributed to damage to the integrity of the rice membrane, leading to increased cell membrane permeability which enhances lipid peroxidation of the membrane, increasing MDA accumulation, reducing the lipid content on the thylakoid membrane, and ultimately resulting in a decreased photosynthetic rate and diminished photosynthetic function in rice [55,56].

## 4. Conclusions

**Figure 7:**
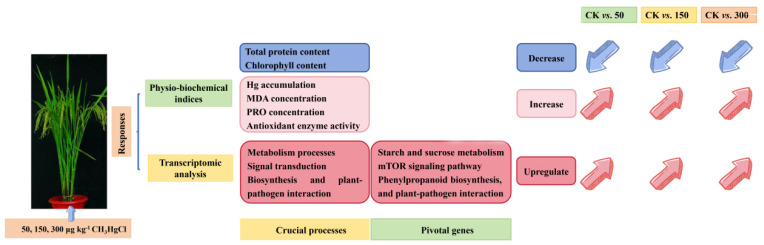
Summary of the transcriptomic and physiological and biochemical features in rice plants in response to different levels of MeHg stress.

In summary, this study provides a comprehensive analysis of the molecular and physiological responses of rice to MeHg stress. A set of DE-miRNAs was identified in rice roots, playing critical roles in regulating metabolic processes, signal transduction, biosynthesis, and plant–pathogen interactions. Among these, several pivotal genes were recognized as essential for the rice response to MeHg toxicity, including those associated with starch and sucrose metabolism (BGIOSGA005631 and BGIOSGA010570), the TOR signaling pathways (BGIOSGA003620), phenylpropanoid biosynthesis (BGIOSGA000436), and defense mechanisms against pathogens (BGIOSGA001663). PPI and miRNA–target gene analyses further revealed genes encoding proteins involved in MeHg stress response pathways, such as jasmonic acid-amido synthetase (BGIOSGA034955), FERONIA-like receptors (BGIOSGA017715, BGIOSGA013411, and BGIOSGA000306), lipoxygenase and peroxidase (osa-miR156b-3p and BGIOSGA013525), and starch synthesis (osa-miR156b-3p and BGIOSGA000900), indicating their potential role in detoxification processes. The involvement of antioxidant enzymes (CAT, POD, and SOD), MDA, and PRO in mitigating ROS induced by MeHg further underscores the importance of these detoxification strategies of rice (Figure 7). The comparative analysis of rice’s response to varying levels of MeHg stress provides valuable insights into its adaptive mechanisms, laying a solid foundation for future research aimed at improving crop resilience to environmental stress.

## Data Availability

The data supporting the findings of this study are available within the article’s Supplementary Materials.

## Supplementary Materials

The following supporting information can be downloaded at https://www.mdpi.com/article/10.3390/toxics13110989/s1, Figure S1: (a) Venn diagram of the common and specific miRNA in roots of rice after exposure to MeHg and in the control. (b) Correlation analysis and (c,d) principal component analysis (PCA) of 3 biological replicates in the con, low, middle, and high groups. Figure S2: (a) Functional annotation of the target genes by prediction software of plants (psRobot). (b) Protein–protein interaction (PPI) and (c) miRNA–target gene correspondence analysis of the commonly responsive differentially expressed miRNAs (DE-miRNAs). Table S1: Quality and concentration of RNA samples. Table S2: List of primer sequences used. Table S3: Quality of sequence data in three replicates for all samples. Table S4: Predicted target genes of some DE-miRNAs. Table S5: Gene Ontology (GO) enrichment analysis of the response DE-miRNAs in roots of rice after exposure to low, middle, and high MHgCl_2_ compared to the control without MeHg (CK). The top 20 highest enriched genes for each treatment are displayed, as well as the biological processes they are linked to. Table S6: Kyoto Encyclopedia of Genes and Genomes (KEGG) pathway enrichment analysis of the response DE-miRNAs in roots of rice after exposure to low, middle, and high MeHg compared to the control without MeHg (CK). The top 20 most enriched KEGG pathways are displayed. Table S7: Key genes screened by the protein–protein interaction (PPI) network analysis. Table S8: The relative gene expression levels [log_2_(fold change)] of the key genes screened by the miRNA–target gene correspondence analysis. Table S9: Key genes screened by the miRNA–target gene correspondence analysis.
