# Supplementary material for: Integrated Transcriptomic and Physiological and Biochemical Analysis Revealed Response Mechanism of Rice (Oryza sativa L.) to Methylmercury Toxicity Stress
Source: Toxics. 2025 Nov 17;13(11):989. doi: 10.3390/toxics13110989 (PMC12656331; doi:10.3390/toxics13110989)
Supplement: Supplementary file 1 [file toxics-13-00989-s001.zip › toxics-3971111-supplementary.pdf]

# Integrated Transcriptomic and physiological and biochemical Analysis Revealed Response Mechanism of Rice (Oryza sativa L.) to Methylmercury Toxicity Stress

**Lin Liu[1*], Kang Wang[1], Shuiting Long[2], Wentao Li[1], ADILI BATUER[1], Lei Wang[3], Tinjia Ma[1], Xiaohang Xu[4], Longchao Liang[5], Md. Habibullah-Al-Mamun[6] and Guangle Qiu[7*]**

[1] College of Civil Engineering and Architecture, Xinjiang University, Urumqi, 830047, China; liulin@xju.edu.cn
[2] Disease Prevention and Control Center of Qiandongnan Miao and Dong Autonomous Prefecture, Kaili, 556099, China; 1078799343@qq.com
[3] College of Environmental Science and Engineering, Institute of Pollution Control and Ecological Safety, Tongji University, Shanghai, 200092, China; celwang@tongji.edu.cn
[4] Key Laboratory of Karst Georesources and Environment, Ministry of Education, College of Resources and Environmental Engineering, Guizhou University, Guiyang, 550025, China; xuxh@gzu.edu.cn
[5] School of Chemistry and Materials Science, Guizhou Normal University, Guiyang, 550025, China; lianglc139@gznu.edu.cn
[6] Department of Fisheries, Faculty of Biological Sciences, University of Dhaka, Dhaka, 1000, Bangladesh; almamunhabib@du.ac.bd
[7] State Key Laboratory of Environmental Geochemistry, Institute of Geochemistry, Chinese Academy of Sciences, Guiyang, 550081, China; qiuguangle@vip.skleg.cn
* Correspondence: Lin Liu, liulin@xju. edu.cn; Tel.:18801227352; Guangle Qiu, qiuguangle@vip.skleg.cn; Tel.:15186996727

## 1.1. Total RNA extraction

To extract total RNA from rice root tips, the following steps were performed to ensure high-quality RNA isolation:

(1) Sample Preparation

Put appropriate amount of rice root tips in a mortar from refrigerator with -80°C and pour in liquid nitrogen and grind them to a powder.

(2) Cell Lysis

Take about 50 mg of powdered tissue samples into a 1.5 ml centrifuge tube pre-chilled with 1 ml of Trizol, shake and mix to lyse well, and leave at room temperature for 10 min, centrifuge at 4 °C, 12,000 g for 5 min.

(3) Phase Separation

Pipette the upper layer of liquid into another centrifuge tube, add (0.2 ml chloroform/1 ml Trizol) pre-cooled chloroform, shake and mix thoroughly, then leave at room temperature for 5 min, centrifuge at 4 °C, 12,000 g for 15 min.

(4) RNA Precipitation

Pipette the supernatant (do not touch the intermediate protein layer) into another

tube, add the same volume of pre-cooled isopropanol as the supernatant, mix the tube upside down, leave it at room temperature for 10 min, centrifuge at 4 °C, 12,000 g for 10 min, and then discard the supernatant.

(5) RNA Washing

Add 1 ml of pre-chilled 75% ethanol to suspend the precipitate, gently invert, and centrifuge at 4 °C and 12000 g for 5 min.

(6) RNA Resuspension

Discard the supernatant, then centrifuge the tube for a few seconds, shake off the residual night hanging on the tube wall, aspirate the residual liquid with a 10 ul gun, dry at room temperature for 3-5 min, then add 20 ul of RNase-Free water to dissolve the RNA, and store the total RNA in a refrigerator at -80°C.

(7) Library Preparation and Sequencing

3μg of total RNA was ligated with sequencing adapters with TruseqTM Small RNA sample prep Kit (Illumina, San Diego, CA, USA). Subsequently, cDNA was synthesized by reverse transcription and amplified with 12 PCR cycles to produce libraries, which was sequenced using Illumina Hiseq X Ten.

**1.2. Bioinformatics analysis**

(1) Raw data preprocessing

Raw sequencing data contain sequencing junction sequences or low-quality reads, and the software Fastx-Toolkit (http://hannonlab.cshl.edu/fastx_toolkit/) was used to filter the raw data to obtain high-quality sequencing data (clean data) to ensure the accuracy of the subsequent bioinformatics analysis. The steps and sequence are as follows:

1) Remove the 3' junction sequences from the reads, and remove the reads without inserted fragments due to junction self-linkage or other reasons;

2) Cut the bases with low sequencing quality at the 3' end (quality value less than 20);

3) Remove the reads containing unknown base N;

4) Removal of reads that are too short (<18nt);

5) Removal of reads that are too long (>32nt).

(2) Identification and differential expression analysis of miRNAs

The 18-32 nt clean sequences obtained after filtering were used to compare the clean reads with the reference genome sequences by Bowtie software (genome version: ASM465v1, downloaded: http://plants.ensembl.org/Oryza_indica/Info/Index), and the reads that matched the reference genome-compliant reads were compared with miRBase to obtain the annotation information of known miRNAs. The reads that did not not match the known miRNAs were annotated using the Rfam database, and the ncRNAs and repetitive sequences, such as ribosomal RNA (rRNA), transfer RNA (tRNA), small intranuclear RNA (snRNA), and small nucleolar RNA (snoRNA), were filtered to obtain the unannotated reads that contained the potential miRNAs. The annotation information of the reads that contained the potential miRNAs was then compared between the Rfam and the miRBase's sRNAs were compared to the reference genome, and their surrounding sequences were intercepted for secondary structure prediction using miRDeep2 software, and new miRNAs were identified based on the prediction results filtered using Dicer enzyme cut site information, energy value and other features. miRNA expression was normalized to TPM (Tags Per Million), and differentially expressed miRNAs (DE-miRNAs) were detected using DESeq2 software, and the screening criteria for DE-miRNAs were:

1) Fold-change ≥ 2, p-value < 0.05 for significant up-regulation;

2) Fold-change ≤ 0.5, p-value < 0.05 for significant down-regulation.

**1.3. Validation of high-throughput sequencing results by real-time fluorescent quantitative PCR (qRT– PCR)**

To validate the high-throughput sequencing results, specific miRNAs were selected for further analysis based on their expression patterns and potential biological relevance.

(1) Selection of Target miRNAs

The following miRNAs were identified from the sequencing data for validation: osa-miR156b-3p, osa-miR319a-3p, and osa-miR390-5p, which were consistently differentially expressed across all three MeHg stress groups. osa-miR393-3p, previously associated with heavy metal stress, and osa-miR396b-5p, predicted as a

potential metallothionein target gene.

(2) RNA Extraction and Reverse Transcription

Total RNA was extracted from the same rice root tip samples used for miRNA sequencing, following the method described in Section 1.1. cDNA synthesis was performed through reverse transcription using specific primers for the target miRNAs.

(3) qRT-PCR Analysis

miRNA expression levels were quantified using qRT-PCR. Expression levels of the target miRNAs in the experimental and control groups were compared to evaluate differential expression.

(4) Internal Reference and Primer Design

U6 small nuclear RNA was used as the internal reference gene to normalize miRNA expression. Primers for qRT-PCR were designed and synthesized by Thermo Fisher Scientific.

**1.4. Determination of and physiological and biochemical indice**

(1) Total protein quantification

Accurately weigh 0.50 g of tissue (root, stem, or leaf) and place it in a 5 mL centrifuge tube. Add 9 volumes of PBS buffer (0.1 mol/L, pH 7.4) to achieve a 1:9 weight-to-volume ratio and prepare a 10% tissue homogenate using mechanical homogenization in an ice-water bath. Centrifuge at 3500 rpm for 10 minutes, and collect the supernatant. For leaves, dilute the supernatant with PBS (1:1) to obtain a 5% homogenate. Mix 0.05 mL of the blank (ultrapure water), standard (0.524 g/L protein solution), or sample with 3 mL of Coomassie Brilliant Blue reagent. Incubate for 10 minutes, measure absorbance at 595 nm using a UV spectrophotometer (1 cm optical path, ultrapure water as a blank), and calculate protein concentration using the appropriate formula.

(2) Proline (PRO) Measurement

Accurately weigh 0.50 g of tissue and prepare a 10% tissue homogenate in PBS under ice-water bath conditions. Centrifuge and collect the supernatant. Mix 0.5 mL of the blank (homogenization medium), standard (5 μg/mL proline solution), or sample with 1 mL each of buffer and color reagent (acidic ninhydrin). Heat in a boiling water

bath for 30 minutes, cool under running water, and measure absorbance at 520 nm (1 cm optical path, ultrapure water as a blank). Calculate the PRO content using the appropriate formula.

(3) Malondialdehyde (MDA) Measurement

Accurately weigh 0.50 g of tissue, homogenize in PBS, and centrifuge to collect the supernatant. Mix 0.2 mL of the blank (anhydrous ethanol), standard (10 nmol/L tetraethoxypropane), or sample with MDA reagent. Incubate in a boiling water bath at 95°C for 40 minutes, cool under running water, and centrifuge at 3500 rpm for 10 minutes. Measure the absorbance of the supernatant at 532 nm (1 cm optical path, ultrapure water as a blank) and calculate MDA content using the appropriate formula.

(4) Peroxidase (POD) Activity

Weigh 0.50 g of tissue and homogenize in PBS. Centrifuge and dilute the supernatant with PBS (1:99) to prepare a 0.1% tissue homogenate. Set up the measurement tube (0.1 mL sample) and control tube (0.1 mL ultrapure water). Add POD reagent to both tubes and incubate at 37°C in a water bath for 30 minutes. Centrifuge at 3500 rpm for 10 minutes, and measure the absorbance of the supernatant at 420 nm (1 cm optical path, ultrapure water as the blank). Calculate POD activity using the respective formula.

(5) Catalase (CAT) Activity

Weigh 0.50 g of tissue and homogenize in PBS. Centrifuge and use the supernatant for analysis. Set up the assay tube (0.2 mL sample at 0.1% concentration) and control tube (0.2 mL ultrapure water). Add CAT reagent and incubate at 37°C for 1 minute. Measure absorbance at 405 nm (ultrapure water as the blank). Calculate CAT activity using the corresponding formula.

(6) Superoxide dismutase (SOD) Activity

Weigh 0.50 g of tissue and homogenize in PBS. Centrifuge and use the supernatant directly for stems. For roots and leaves, dilute the supernatant 1:4 with PBS to prepare a 2% tissue homogenate. Set up a blank tube (ultrapure water) and test tube (0.05 mL sample). Add SOD reagent, mix, and incubate in a 37°C water bath for 40 minutes. Add 2 mL of color developer, mix well, and leave at room temperature for 10 minutes.

Measure absorbance at 550 nm (1 cm optical path, ultrapure water as the blank). Calculate SOD activity using the appropriate formula.

(7) Chlorophyll (CHL) Content Measurement

Fresh rice leaves (midvein removed) and stem tissues were washed with ultrapure water, blotted dry, weighed to approximately 0.1 g, cut into small pieces, and placed in a 5 mL centrifuge tube. After adding 1 mL ultrapure water and 50 mg of chlorophyll reagent, the tissue was homogenized under light-proof conditions. The volume was adjusted to 5 mL with extraction solution (anhydrous ethanol and acetone in a 1:2 volume ratio), and the mixture was extracted under light-proof conditions for ~3 hours until the tissue turned white. Stems were centrifuged at 4000 rpm for 10 minutes, and the supernatant was used directly. Leaf supernatant was diluted 10-fold with extraction solution. Absorbance was measured at 645 nm and 663 nm using the extraction solution as a blank. Chlorophyll a, chlorophyll b, and total chlorophyll content were calculated using specific formulas.

# Figures

**Figure. S1.** (a) Venn diagram of the common and specific miRNA in roots of rice exposure to MeHg and in the control. (b) correlation analysis and (c)(d) principal component analysis (PCA) of 3 biological replicates in the Con, Low, Middle, and High groups.

**Figure. S2.** (a) Functional annotation of the target genes by prediction software of plants (psRobot). (b) Protein−protein interaction (PPI) and (c) miRNA-target gene correspondence analysis of the common response differentially expressed miRNAs (DE-miRNAs).

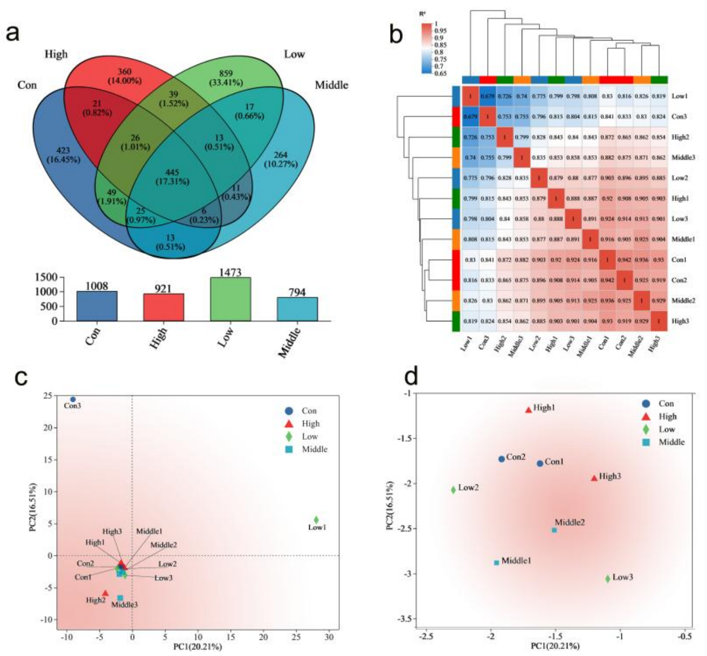


**Figure. S1.** (a) Venn diagram of the common and specific miRNA in roots of rice exposure to MeHg and in the control. (b) correlation analysis and (c)(d) principal component analysis (PCA) of 3 biological replicates in the Con, Low, Middle, and High groups.

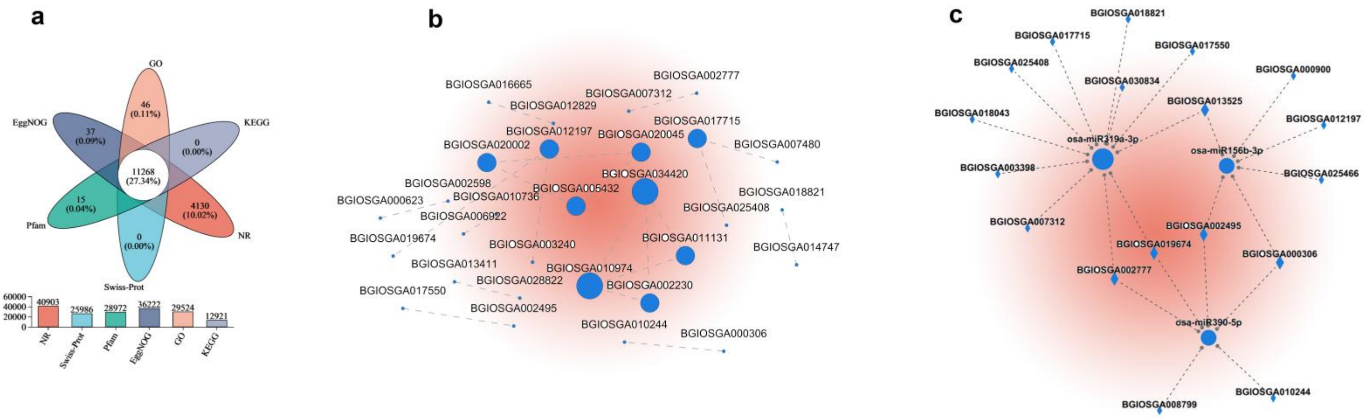


**Figure. S2.** (a) Functional annotation of the target genes by prediction software of plants (psRobot). (b) Protein–protein interaction (PPI) and (c) miRNA-target gene correspondence analysis of the common response differentially expressed miRNAs (DE-miRNAs).

# Tables

**Table S1** The quality and concentration of RNA samples.

**Table S2** List of primer sequences used.

**Table S3** The quality of sequence data in four replicates for all samples.

**Table S4** The predicted target gene of some DE-miRNAs.

**Table S5** Gene Ontology (GO) enrichment analysis of the response DE-miRNAs in roots of rice exposure to low, middle, and high $MHgCl_2$ compared to the control without MeHg (CK). The top 20 highest enriched genes for each treatment, as well as the biological processes they are linked to, are displayed.

**Table S6** Kyoto Encyclopedia of Genes and Genomes (KEGG) pathway enrichment analysis of the response DE-miRNAs in roots of rice exposure to low, middle, and high MeHg compared to the control without MeHg (CK). The top 20 most enriched KEGG pathways are displayed.

**Table S7** Key genes screened by the protein–protein interaction (PPI) network analysis.

**Table S8** The relative gene expression levels [$\log_2$(fold change)] of the key genes screened by the miRNA-target gene correspondence analysis.

**Table S9** Key genes screened by the miRNA-target gene correspondence analysis.

**Table S1** The quality and concentration of RNA samples.

| **Samples** | **RNI** | **28S/18S** | **Concentration(ng/μL)** | **OD260/OD280** | **OD260/OD230** |
|---|---|---|---|---|---|
| Con1 | 9.30 | 2.3 | 221.70 | 1.99 | 1.88 |
| Con2 | 9.00 | 2.3 | 150.30 | 1.92 | 1.57 |
| Con3 | 9.10 | 2.3 | 156.40 | 1.91 | 1.55 |
| Low1 | 9.50 | 1.8 | 155.40 | 1.92 | 1.65 |
| Low2 | 9.20 | 1.8 | 150.00 | 1.90 | 1.62 |
| Low3 | 8.90 | 2.1 | 151.70 | 1.97 | 1.73 |
| Middle1 | 8.40 | 2.2 | 339.50 | 2.05 | 2.02 |
| Middle2 | 9.10 | 2.5 | 324.80 | 2.05 | 2.07 |
| Middle3 | 9.20 | 2.7 | 189.50 | 1.96 | 1.74 |
| High1 | 9.30 | 2.4 | 151.20 | 1.98 | 1.69 |
| High2 | 9.20 | 2.4 | 153.30 | 2.01 | 1.75 |
| High3 | 9.00 | 2.5 | 161.00 | 2.02 | 1.82 |

Notes: RNA integrity number (RIN) value was used to measure the quality of RNA sample. RIN value ≥ 7.0 indicates a high quality RNA.

**Table S2** List of primer sequences used.

| Gene ID | Amplified sequence (5’-3’) | Length (bp) |
|---|---|---|
| Osa-miR396b-5p | F：CGCGTTCCACAGCTTTCTT | 60 |
| | R：AGTGCAGGGTCCGAGGTATT | |
| Osa-miR393b-3p | F：CGCGTCAGTGCAATCCCTT | 60 |
| | R：AGTGCAGGGTCCGAGGTATT | |
| Osa-miR390-5p | F：CGCGAAGCTCAGGAGGGAT | 60 |
| | R：AGTGCAGGGTCCGAGGTATT | |
| Osa-miR319a-3p | F：CGACTGGATGACGCGGG | 60 |
| | R：AGTGCAGGGTCCGAGGTATT | |
| Osa-miR156b-3p | F：GCGCGGCTCACTCTCTATCT | 60 |
| | R：AGTGCAGGGTCCGAGGTATT | |

| | | | |
|---|---|---|---|
| U6 (internal standard primers) | F: TTGGGACGATACACAGAAAATTAG | 61 |
| | R: GATTTGTGCGTGTCATTCCTGTC | |

Notes: F:Forward Primer, R:Reverse Primer

**Table S3** The quality of sequence data in three replicates for all samples.

| **Samples** | **Total reads** | **Clean reads** | **Clean bases** | **Q30 (%)** | **Mapped reads** |
|---|---|---|---|---|---|
| Con1 | 17466785 | 15696965 | 331002537 | 97.3 | 2823234 |
| Con2 | 18820634 | 16593484 | 338953198 | 97.23 | 2352982 |
| Con3 | 17362229 | 15247858 | 326202659 | 96.91 | 3033326 |
| Low1 | 23340244 | 20311397 | 431733475 | 97.42 | 3496656 |
| Low2 | 22247958 | 19316821 | 395763285 | 97.33 | 3117942 |
| Low3 | 18567223 | 16159023 | 313006800 | 97.4 | 2262950 |
| Middle1 | 16091972 | 14293360 | 309292667 | 97.31 | 4685315 |
| Middle2 | 17884570 | 16628353 | 377614621 | 97.12 | 2776605 |
| Middle3 | 18673790 | 17206695 | 388264888 | 97 | 3187623 |
| High1 | 19898806 | 17540166 | 375782209 | 97.34 | 3166584 |
| High2 | 20213175 | 18270717 | 393139178 | 97.37 | 3507366 |
| High3 | 16137289 | 14282648 | 302561091 | 97.24 | 2512743 |

Notes: Q30 (%) indicates the percentage of the bases with a sequencing accuracy of 99.9% for each base.

**Table S4** The predicted target gene of some differentially expressed miRNA.

| Differentially expressed miRNA and its sequence | ID and functional annotation of target gene in genome |
|---|---|
| osa-miR1868 UCACGGAAAACG AGGGAGCAGCCA | BGIOSGA017979\|Tubby-like F-box protein<br>BGIOSGA000007\|mitochondrial<br>BGIOSGA022588\|Casparian strip membrane protein 3<br>BGIOSGA014173\|Inositol-pentakisphosphate 2-kinase IPK1<br>BGIOSGA002115\|Endoglucanase |

| | |
|---|---|
| | BGIOSGA008993\|Endoglucanase<br>BGIOSGA013505\|Dihydrolipoyl dehydrogenase<br>BGIOSGA002422\|Probable zinc metalloprotease EGY2<br>BGIOSGA028515\|Dirigent protein<br>BGIOSGA021512\|Dirigent protein<br>BGIOSGA023732\|Probable E3 ubiquitin-protein ligase BAH1-like 1 |
| osa-miR395g<br>GUGAAGUGUUUG<br>GGGGAACUC | BGIOSGA015284\|ATP-dependent DNA helicase<br>BGIOSGA036199\|Auxin response factorAuxin response factor<br>BGIOSGA002229\|Malic enzyme<br>BGIOSGA024510\|Metal transporter |
| osa-miR396b-5p<br>UUCCACAGCUUU<br>CUUGAACUG | BGIOSGA011835\|ABC transporter C family member 13<br>BGIOSGA026730\|Heat shock protein 81-1<br>BGIOSGA019338\|Metallothionein-like protein 3B<br>BGIOSGA006243\|MLO-like protein<br>BGIOSGA019132\|Peroxidase |
| osa-miR393b-3p<br>UCAGUGCAAUCC<br>CUUUGGAAU | BGIOSGA019973\|Protein YIPF<br>BGIOSGA015280\|ATP-dependent DNA helicase<br>BGIOSGA036014\|Lipoxygenase<br>BGIOSGA031324\|NADH dehydrogenase<br>BGIOSGA016215\|Probable magnesium transporter |
| osa-miR390-5p<br>AAGCUCAGGAGG<br>GAUAGCGCC | BGIOSGA027394\|Glucose-6-phosphate 1-epimerase<br>BGIOSGA029279\|NADPH--cytochrome P450 reductase<br>BGIOSGA023548\|Mitogen-activated protein kinase<br>BGIOSGA034888\|Pectinesterase<br>BGIOSGA012582\| Potassium transporter |

**Table S5** Gene Ontology (GO) enrichment analysis of the response DE-miRNAs in roots of rice exposure to low, middle, and high $MHgCl_2$ compared to the control without MeHg (CK). The top 20 highest enriched genes for each treatment, as well as the biological processes they are linked to, are displayed.

| con-low | | | | | con-middle | | | | | con-high | | | | |
|---|---|---|---|---|---|---|---|---|---|---|---|---|---|---|
| GO ID | Term Type | Functional description | Number of enriched genes | Padjust | GO ID | Term Type | Functional description | Number of enriche d genes | Padjust | GO ID | Term Type | Functional description | Number of enriche d genes | Padjust |
| GO:0022414 | BP | reproductive process | 437 | 2.0E-7 | GO:0008037 | BP | cell recognition | 116 | 9.1E-4 | GO:0045930 | BP | negative regulation of mitotic cell cycle | 20 | 1.9E-3 |
| GO:0006468 | BP | protein phosphorylation | 1161 | 2.0E-7 | GO:0048544 | BP | recognition of pollen | 116 | 9.1E-4 | GO:0022402 | BP | cell cycle process | 89 | 1.9E-3 |
| GO:0016310 | BP | phosphorylation | 1438 | 2.0E-7 | GO:0051276 | BP | chromosome organization | 142 | 9.1E-4 | GO:0006468 | BP | protein phosphorylation | 566 | 1.9E-3 |
| GO:0006796 | BP | phosphate-containing compound metabolic process | 1810 | 2.0E-7 | GO:0051128 | BP | regulation of cellular component organization | 162 | 9.1E-4 | GO:0016310 | BP | phosphorylation | 691 | 1.9E-3 |
| GO:0006793 | BP | phosphorus metabolic process | 1819 | 2.0E-7 | GO:0022402 | BP | cell cycle process | 169 | 9.1E-4 | GO:0006796 | BP | phosphate-containing compound metabolic process | 862 | 1.9E-3 |
| GO:0044267 | BP | cellular protein metabolic process | 2203 | 2.0E-7 | GO:0007017 | BP | microtubule-based process | 123 | 9.1E-4 | GO:0043412 | BP | macromolecule modification | 960 | 1.9E-3 |
| GO:0044702 | BP | single organism reproductive process | 381 | 2.0E-7 | GO:0044702 | BP | single organism reproductive process | 412 | 9.1E-4 | GO:0006793 | BP | phosphorus metabolic process | 867 | 1.9E-3 |

| | | | | | | | | | | | | | | |
|---|---|---|---|---|---|---|---|---|---|---|---|---|---|---|
| GO:0043412 | BP | macromolecule modification | 2042 | 2.0E-7 | GO:1902589 | BP | single-organism organelle organization | 335 | 9.1E-4 | GO:0006464 | BP | cellular protein modification process | 907 | 1.9E-3 |
| GO:0006464 | BP | cellular protein modification process | 1900 | 2.0E-7 | GO:0022414 | BP | reproductive process | 473 | 9.1E-4 | GO:0036211 | BP | protein modification process | 907 | 1.9E-3 |
| GO:0036211 | BP | protein modification process | 1900 | 2.0E-7 | GO:0006996 | BP | organelle organization | 589 | 9.1E-4 | GO:0044260 | BP | cellular macromolecule metabolic process | 1745 | 1.9E-3 |
| GO:0019538 | BP | protein metabolic process | 2699 | 2.0E-7 | GO:0044431 | CC | Golgi apparatus part | 252 | 9.1E-4 | GO:0043170 | BP | macromolecule metabolic process | 1992 | 1.9E-3 |
| GO:0032403 | MF | protein complex binding | 135 | 2.0E-7 | GO:0015631 | MF | tubulin binding | 99 | 9.1E-4 | GO:0008037 | BP | cell recognition | 59 | 1.9E-3 |
| GO:0008017 | MF | microtubule binding | 97 | 2.0E-7 | GO:0008017 | MF | microtubule binding | 94 | 9.1E-4 | GO:0048544 | BP | recognition of pollen | 59 | 1.9E-3 |
| GO:0008092 | MF | cytoskeletal protein binding | 164 | 2.0E-7 | GO:0008092 | MF | cytoskeletal protein binding | 169 | 9.1E-4 | GO:0005886 | CC | plasma membrane | 375 | 1.9E-3 |
| GO:0015631 | MF | tubulin binding | 101 | 2.0E-7 | GO:0004386 | MF | helicase activity | 152 | 9.1E-4 | GO:0009506 | CC | plasmodesma | 109 | 1.9E-3 |
| GO:0043531 | MF | ADP binding | 442 | 2.0E-7 | GO:0022804 | MF | active transmembrane transporter activity | 404 | 9.1E-4 | GO:0030054 | CC | cell junction | 109 | 1.9E-3 |
| GO:0016887 | MF | ATPase activity | 456 | 2.0E-7 | GO:0043531 | MF | ADP binding | 498 | 9.1E-4 | GO:0005911 | CC | cell-cell junction | 109 | 1.9E-3 |
| GO:0017111 | MF | nucleoside-triphosphatase activity | 639 | 2.0E-7 | GO:0042623 | MF | ATPase activity, coupled | 356 | 9.1E-4 | GO:0008092 | MF | cytoskeletal protein binding | 95 | 1.9E-3 |
| GO:0016817 | MF | hydrolase activity, acting on acid anhydrides | 675 | 2.0E-7 | GO:0016887 | MF | ATPase activity | 486 | 9.1E-4 | GO:0032403 | MF | protein complex binding | 74 | 1.9E-3 |

| | | | | | | | | | | | | | |
|---|---|---|---|---|---|---|---|---|---|---|---|---|---|
| GO:0016818 | MF | hydrolase activity, in phosphorus-containing anhydrides | 669 | 2.0E-7 | GO:0017111 | MF | nucleoside-triphosphatase activity | 683 | 9.1E-4 | GO:0044877 | MF | macromolecular complex binding | 117 | 1.9E-3 |

**Table S6** Kyoto Encyclopedia of Genes and Genomes (KEGG) pathway enrichment analysis of the response DE-miRNAs in roots of rice exposure to low, middle, and high MeHg compared to the control without MeHg (CK). The top 20 most enriched KEGG pathways are displayed.

| con-low | | | con-middle | | | con-high | | |
|---|---|---|---|---|---|---|---|---|
| Functional description | Pathway id | Number of enriched genes | Functional description | Pathway id | Number of enriched genes | Functional description | Pathway id | Number of enriched genes |
| Starch and sucrose metabolism | map00500 | 144 | Starch and sucrose metabolism | map00500 | 162 | Ubiquitin mediated proteolysis | map04120 | 56 |
| Homologous recombination | map03440 | 71 | MicroRNAs in cancer | map05206 | 63 | Insulin resistance | map04931 | 26 |
| alpha-Linolenic acid metabolism | map00592 | 48 | FoxO signaling pathway | map04068 | 62 | MAPK signaling pathway - fly | map04013 | 24 |
| MicroRNAs in cancer | map05206 | 56 | Glycerophospholipid metabolism | map00564 | 100 | p53 signaling pathway | map04115 | 20 |
| Linoleic acid metabolism | map00591 | 28 | Adipocytokine signaling pathway | map04920 | 45 | RNA degradation | map03018 | 48 |
| Ribosome biogenesis in eukaryotes | map03008 | 77 | Phenylpropanoid biosynthesis | map00940 | 208 | Antifolate resistance | map01523 | 12 |
| Bile secretion | map04976 | 31 | Cyanoamino acid metabolism | map00460 | 61 | Adipocytokine signaling pathway | map04920 | 22 |

| | | | | | | | | |
|---|---|---|---|---|---|---|---|---|
| Glycosylphosphatidylinositol (GPI)-anchor biosynthesis | map00563 | 20 | Ether lipid metabolism | map00565 | 30 | Choline metabolism in cancer | map05231 | 23 |
| HIF-1 signaling pathway | map04066 | 32 | Insulin resistance | map04931 | 53 | Gastric cancer | map05226 | 20 |
| Insulin resistance | map04931 | 47 | Proteasome | map03050 | 55 | Phospholipase D signaling pathway | map04072 | 20 |
| Adipocytokine signaling pathway | map04920 | 37 | Hypertrophic cardiomyopathy (HCM) | map05410 | 28 | Regulation of actin cytoskeleton | map04810 | 21 |
| Protein digestion and absorption | map04974 | 10 | Bile secretion | map04976 | 33 | Endocrine resistance | map01522 | 6 |
| mTOR signaling pathway | map04150 | 50 | alpha-Linolenic acid metabolism | map00592 | 49 | Plant-pathogen interaction | map04626 | 81 |
| Toll and Imd signaling pathway | map04624 | 149 | Homologous recombination | map03440 | 74 | ErbB signaling pathway | map04012 | 5 |
| Inositol phosphate metabolism | map00562 | 45 | Longevity regulating pathway | map04211 | 60 | Glycosphingolipid biosynthesis - ganglio series | map00604 | 5 |
| Chagas disease (American trypanosomiasis) | map05142 | 155 | Meiosis - yeast | map04113 | 60 | Fc gamma R-mediated phagocytosis | map04666 | 21 |
| Gastric cancer | map05226 | 35 | Gastric cancer | map05226 | 41 | Inositol phosphate metabolism | map00562 | 25 |
| Cell cycle - yeast | map04111 | 79 | Hippo signaling pathway - multiple species | map04392 | 9 | Bacterial secretion system | map03070 | 11 |

| | | | | | | | | |
|---|---|---|---|---|---|---|---|---|
| Carotenoid biosynthesis | map00906 | 28 | Cell cycle - yeast | map04111 | 91 | Other glycan degradation | map00511 | 10 |
| Toll-like receptor signaling pathway | map04620 | 147 | Protein digestion and absorption | map04974 | 11 | Colorectal cancer | map05210 | 13 |

**Table S7** Key genes screened by the protein–protein interaction (PPI) network analysis.

| Node1 | Node2 | node1_accession_id | node2_accession_id | Annotation | KEGG_Orthology | Group |
|---|---|---|---|---|---|---|
| OsI_20654 | OsI_25407 | BGIOSGA017715 | BGIOSGA025408 | Ribosome biogenesis in eukaryotes | K14558 | biosynthesis |
| OsI_20654 | OsI_05761 | BGIOSGA017715 | BGIOSGA007480 | Ribosome biogenesis in eukaryotes | K14558 | biosynthesis |
| OsI_13200 | OsI_29536 | BGIOSGA013411 | BGIOSGA028822 | Cell cycle - yeast | K08866 | - |
| OsI_04829 | OsI_12615 | BGIOSGA000306 | BGIOSGA010244 | Cell cycle - yeast | K06674 | - |
| OsI_18432 | OsI_16588 | BGIOSGA018821 | BGIOSGA014747 | Basal transcription factors,Nucleotide excision repair | K10844 | - |
| OsI_05457 | OsI_00390 | BGIOSGA007312 | BGIOSGA002777 | Ubiquitin mediated proteolysis | K10590 | metabolism processes |
| OsI_01354 | OsI_10676 | BGIOSGA003240 | BGIOSGA012197 | Ubiquitin mediated proteolysis | K10590 | metabolism processes |
| OsI_10676 | OsI_19594 | BGIOSGA012197 | BGIOSGA019674 | Ubiquitin mediated proteolysis | K03348 | metabolism processes |
| OsI_11967 | OsI_16437 | BGIOSGA012829 | BGIOSGA016665 | Pyrimidine metabolism, RNA polymerase, Purine metabolism, Cytosolic DNA-sensing pathway | K03018 | metabolism processes |
| OsI_06283 | OsI_11561 | BGIOSGA006922 | BGIOSGA010736 | RNA transport, the transport of RNA molecules from the nucleus to the cytoplasm | K14310 | signal transduction |

| | | | | | | |
|---|---|---|---|---|---|---|
| OsI_21011 | OsI_00169 | BGIOSGA017550 | BGIOSGA002495 | RNA transport, the transport of RNA molecules from the nucleus to the cytoplasm | K03251 | signal transduction |

**Table S8** The relative gene expression levels [$log_2$(fold change)] of the key genes screened by the miRNA-target gene correspondence analysis.

| miRNA | $Log_2FC$ (CK *vs.* Low) | Regulated | $Log_2FC$ (CK *vs.* Middle) | Regulated | $Log_2FC$ (CK *vs.* High) | Regulated |
|---|---|---|---|---|---|---|
| osa-miR156b-3p | -1.28 | down | -2.28 | down | -1.16 | down |
| osa-miR319a-3p | 1.12 | up | 1.39 | up | 1.22 | up |
| osa-miR390-5p | 1.34 | up | 1.5 | up | 1.11 | up |

**Table S9** Key genes screened by the miRNA-target gene correspondence analysis.

| miRNA | Target gene id | Annotation | KEGG_Orthology | Group |
|---|---|---|---|---|
| osa-miR156b-3p | BGIOSGA013525 | Lipoxygenase, prevent cell lipid peroxidation and resist stress | K15718 | metabolism processes |
| osa-miR156b-3p | BGIOSGA000900 | Starch synthase, chloroplastic/amyloplastic, involved in host defense response toward susceptibility against Rhizoctonia solani in rice | - | metabolism processes |
| osa-miR319a-3p | BGIOSGA030834 | Chlorophyll a-b binding protein, chloroplastic, involved in host defense response toward susceptibility against Rhizoctonia solani in rice | K08908 | metabolism processes |
| osa-miR319a-3p | BGIOSGA003398 | Peroxidase, protect cells from the toxic effects of peroxide | K00430 | biosynthesis |

| | | | | |
|---|---|---|---|---|
| osa-miR390-5p | BGIOSGA008799 | Protein detoxification, involved in host defense response toward susceptibility against Rhizoctonia solani in rice | K03327 | biosynthesis |
| osa-miR319a-3p | BGIOSGA018043 | MLO-like protein, in response to infection by the rice blast fungus | K08472 | signal transduction |
| osa-miR156b-3p | BGIOSGA025466 | Cellulose synthase, as a signaling molecule in plant defense | K10999 | signal transduction |
